# A case of esophageal perforation after intraoperative transesophageal echocardiography in a patient with a giant left atrium: unexpectedly large distortion of the esophagus revealed on retrospectively constructed three-dimensional imaging

**DOI:** 10.1186/s40981-019-0243-0

**Published:** 2019-03-15

**Authors:** Aya Kimura, Takashi Mori, Yuki Kihara, Chie Watanabe, Katsuaki Tanaka, Tokuhiro Yamada, Atushi Yoshida, Joji Kawabe, Yoshito Sakon, Toshihiko Sibata, Kiyonobu Nishikawa

**Affiliations:** 1 Department of Anesthesiology, Osaka City University Graduate School of Medicine, 1-5-7, Asahi-Machi, Abeno-Ku, Osaka, 545-8586 Japan; 2 Department of Nuclear Medicine, Osaka City University Graduate School of Medicine, Osaka, Japan; 3 Department of Cardiovascular Surgery, Osaka City University Graduate School of Medicine, Osaka, Japan

**Keywords:** Esophageal perforation, Transesophageal echocardiography, Mitral stenosis, Giant left atrium

## Abstract

**Background:**

Esophageal perforation is a rare but serious complication of transesophageal echocardiography (TEE). An enlarged left atrium (LA), which is commonly associated with mitral stenosis (MS), is an under-recognized risk factor for esophageal perforation after intraoperative TEE.

**Case presentation:**

We describe a case of TEE-induced esophageal perforation after cardiac surgery in a 79-year-old woman with a giant LA due to MS. Esophageal perforation was detected on postoperative day 6. After surgical repair, the patient gradually recovered with prolonged conservative treatment. Retrospectively constructed three-dimensional chest computed tomography images revealed an unusually distorted esophagus that was possibly vulnerable to injury.

**Conclusion:**

A giant LA can markedly distort the esophagus. It should be recognized as a risk factor for TEE-induced esophageal perforation.

## Background

Transesophageal echocardiography (TEE)-related esophageal perforation is a life-threatening complication with an extremely low incidence (0.03 to 0.09%) [1–3]. Preoperative risk evaluation is recommended to avoid perforation, and early detection would be crucial for improving outcomes [4, 5]. We report a case of esophageal perforation after cardiac surgery in a patient with an enlarged left atrium (LA). Enlarged LA is a risk factor for TEE-associated esophageal perforation [4]; however, it seems to be under-recognized. We retrospectively evaluated three-dimensional (3D) images of the esophagus and found unusual distortion caused by a giant LA.

## Case presentation

Written informed consent was obtained from the patient for the publication of this case report. A 79-year-old, 42-kg woman with severe mitral stenosis (MS) was admitted for mitral valve replacement. She had a history of hypertension, atrial fibrillation, and cerebral infarction. There were no symptoms or history of gastrointestinal disease. Chest radiography showed cardiomegaly with a heart-to-chest ratio of 0.68. Preoperative transthoracic echocardiography and TEE showed severe MS, LA enlargement, and a small left ventricular (LV) cavity (LA diameter 98 × 69 mm, LA volume index 191 ml/m^2^, mitral valve area 0.86 cm^2^, LV diastolic/systolic diameter 38/19 mm, and ejection fraction 62%).

**Fig. 1:**
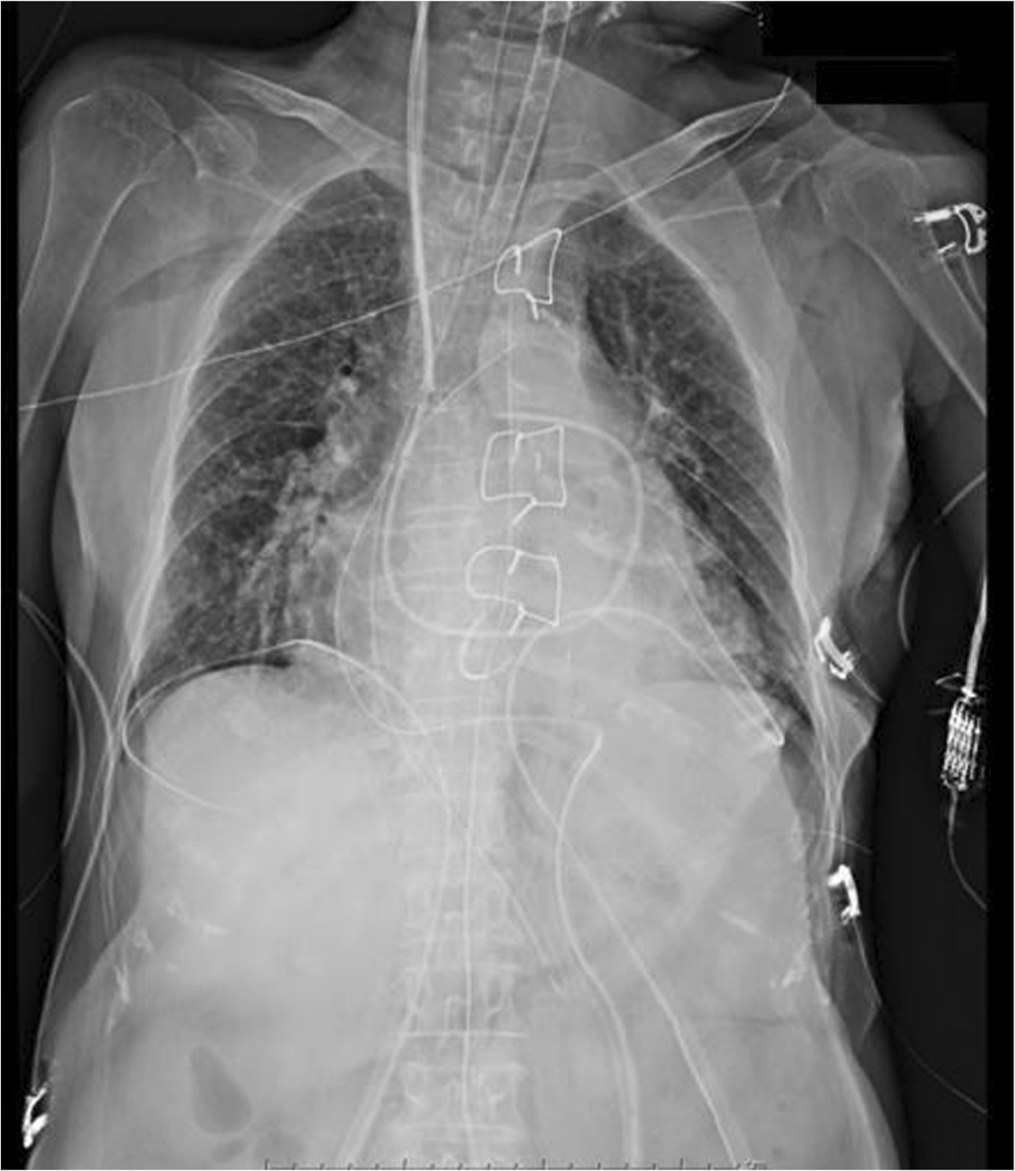
Chest radiography just after the cardiac surgery. The course and the tip position of the NG tube were unusually shifted to the right

After peripheral venous and arterial cannulation, general anesthesia was induced with 300 μg fentanyl, 5 mg midazolam, and 40 mg rocuronium. After intubation, a TEE probe (General Electric, Fairfield, CT, USA) was inserted into the esophagus without any resistance. A central venous catheter and a Swan-Ganz catheter were inserted via the right internal jugular vein. During the initial TEE examination, we were able to advance the probe smoothly but experienced some difficulty in consistently visualizing the transgastric short-axis view. The view was occasionally visualized during repetitive probe advancement below the lower esophagus. TEE was used intermittently during the surgery to avoid mechanical and thermal trauma. The mitral valve was successfully replaced. During weaning from cardiopulmonary bypass (CPB), a small LV rupture was found on the anterior wall. Full-flow CPB was restarted and the rupture was repaired. Intra-aortic balloon pumping (IABP) was introduced during the second attempt to wean from CPB in addition to maximum catecholamine support. During TEE examinations after CPB was weaned off, it was again difficult to obtain images in the transgastric short-axis view. The operative time was 519 min. The TEE probe was removed, and a nasogastric (NG) tube was smoothly inserted. Only a small amount of gastric juice-like fluid drained from the NG tube. Chest radiography just after surgery showed that the course of the NG tube was unusually shifted slightly to the right and running down to the abdomen (Fig. 1). Then, we immediately checked the chest computed tomography (CT) which was performed before the operation. A rightward shift of the esophageal wall was identified from the CT, which lead to the idea that the NG tube was in the correct position.

The patient was transferred to the intensive care unit (ICU). Her cardiovascular condition improved steadily. On postoperative day (POD) 3, she was weaned from IABP support. She was extubated on the following day. Almost no fluid drained from the NG tube since placement; however, bubble sounds with air instillation were confirmed on auscultation with a stethoscope over the epigastrium. Enteral nutrition through the NG tube was started on POD 5. She complained of abdominal pain soon after the initiation of tube feeding. Her respiratory and hemodynamic condition subsequently worsened. She developed hypoxemia and severe hypotension. Mechanical ventilation after orotracheal intubation and hemodynamic support with catecholamine infusion were reintroduced. Abdominal radiography on the same day revealed that the tip of the NG tube did not overlap with the gastric gas pattern; migration of the tube out of the esophagus was suspected. Emergent CT detected esophageal perforation just proximal to the esophagogastric junction (EGJ) and migration of the NG tube through the perforation into the mediastinum and abdominal cavity. These findings were confirmed with subsequent upper gastrointestinal endoscopy. The perforation was repaired through emergency laparotomy. Endoscopic and surgical findings were consistent; the perforation was approximately 2 cm in size and 5 cm above the EGJ. There was no violation of the pleura, distinct areas of local ischemia, or obvious thinning of the esophageal wall. After surgical repair, the patient was treated conservatively in the ICU. Tracheostomy was performed on POD 12. A barium study showed a small amount of leakage that persisted for approximately 2 months from the repair, which finally resolved on POD 72. The patient’s condition gradually improved. She was transferred to another care facility on POD 125.

## Discussion

TEE-induced esophageal perforation can occur even when TEE examination is smooth [4, 6–11]. In the present case, probe insertion was smooth, but we encountered some difficulties in obtaining the transgastric short-axis view during surgery. Although NG tube-induced perforation could not be ruled out completely, it was reasonable to think that the perforation was caused by TEE probe based on our observations, the relatively larger perforation size, and the unusually difficult TEE manipulation.

Esophageal perforation during intraoperative TEE is mostly caused by direct mechanical trauma related to probe insertion and manipulation [4]. Prolonged, continuous pressure and thermal energy from a probe can also damage esophageal tissue, resulting in indirect mechanical trauma. Predisposing factors include anatomical and pathological changes in the esophagus, including strictures, diverticula, tumors, varices, distortions due to an enlarged LA or thoracic aortic aneurysm, pathologic tissue fragility due to chronic steroid therapy or prior chest radiation, prior esophageal surgery, and decreased local circulation due to atherosclerosis [1, 4, 5]. A giant LA was the most significant risk factor in the present case.

**Fig. 2:**
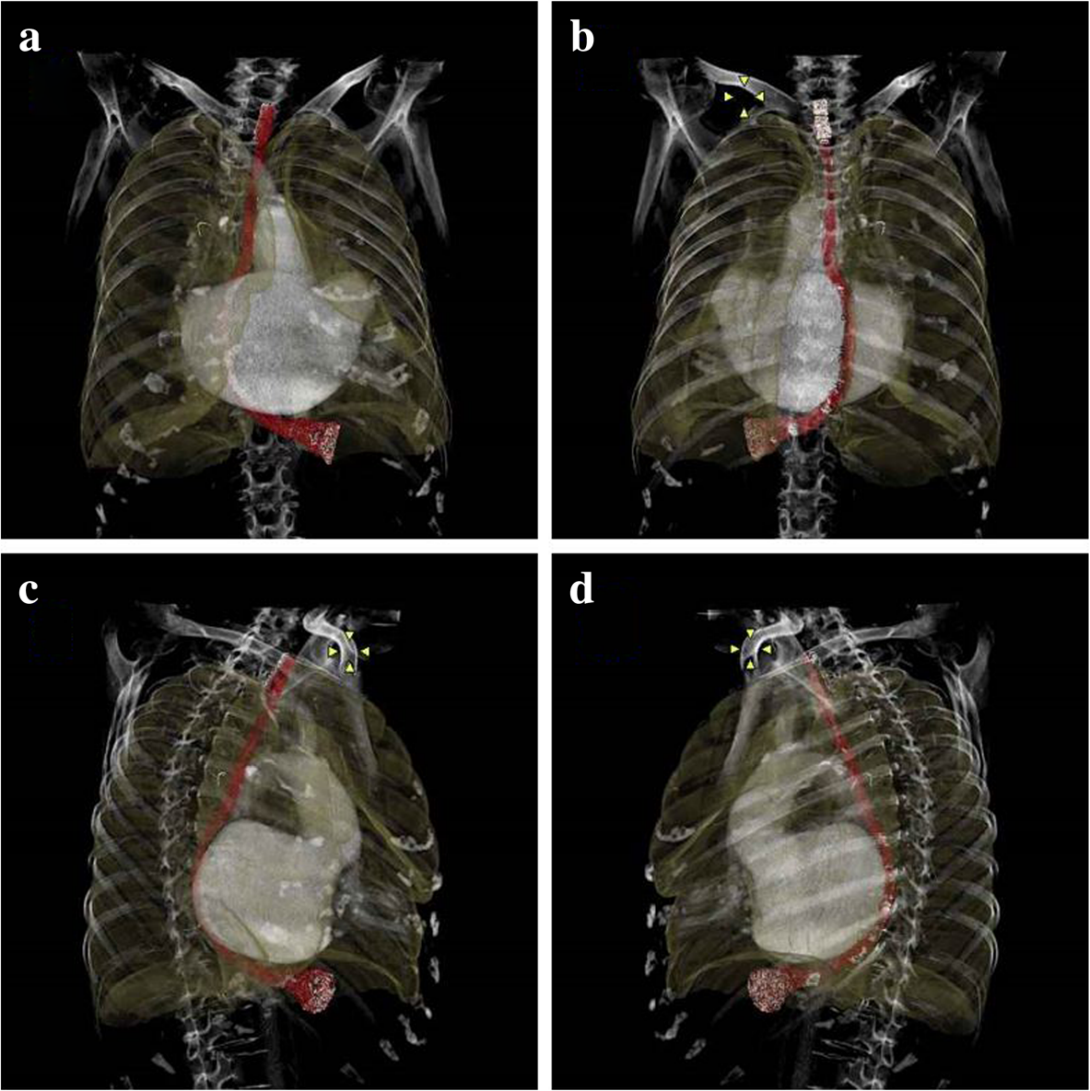
Three-dimensional images of the esophagus. **a** Anterior view. **b** Posterior view. **c** Right lateral oblique view. **d** Left lateral oblique view. The course of the esophagus is shown in red. The esophagus was compressed and distorted by the enlarged left atrium

Gross cardiomegaly characteristic of giant LA can distort and cause thinning of the esophageal wall [4, 6]. Massey et al. have reported iatrogenic esophageal perforation in a patient with a grossly enlarged LA that was 16.5 cm in diameter [6]. However, the manner by which a giant LA distorts the esophagus is yet to be determined. We retrospectively constructed 3D images of the esophagus from the chest CT taken 2 months before the operation using a Synapse Vincent™ (Fuji Film, Tokyo, Japan). The images clearly indicated that the enlarged LA markedly compressed the esophagus rightward and backward, resulting in a twisted distortion (Fig. 2). The figure also showed that the most narrowing part was the middle esophagus. However, the perforation occurred below the middle esophagus. It might be postulated that undue strain by the TEE probe could be placed on the lower esophagus as enlarged LA caused the bend of the esophagus at almost right angle.

We determined the course of the esophagus based on chest CT in patients with MS who underwent cardiac surgery at our hospital during the last 7 years (from 2012 to 2018). Among 33 patients, two patients, including the present patient, had a twisted distortion. Thus, the twisted esophagus in the present patient seemed to be unusual, but it should be noted that an enlarged LA may infrequently cause severe distortion.

Early detection of esophageal perforation is important for improving outcomes. However, detection is often delayed, especially with intraoperative TEE [4, 6–11]. A systematic review reported that the median time until detection was 2.5 days [4]. The presenting symptoms varied by case, including hypotension, shock, fever, dyspnea, hypoxemia, leukocytosis, chest pain, pericardial or pleural effusion, pneumoperitoneum, pneumothorax, and pneumomediastinum [6–11]. There were several clinical findings that might be related to esophageal perforation, such as difficulty in visualizing the transgastric short-axis view, unusual NG tube position on postoperative chest radiography, and no fluid drainage from the NG tube in the ICU. Careful check of the chest X-ray and checking the pH of the fluid from NG tube after the cardiac surgery might be a hint to suspect esophageal perforation and might have led earlier detection.

For the prevention of esophageal perforation, it is important to recognize the risk factors listed above (the second paragraph in the “Discussion” section). Esophageal strictures, tumor, diverticula, tracheoesophageal fistula, post-esophageal surgery, and esophageal trauma are absolute contraindications to TEE [5, 12]. Patients with relative contraindications such as an enlarged LA should be evaluated on a case-by-case basis and appropriate precautions should be considered. [5]. The appropriate precautions include not advancing probe past the mid-esophagus, limiting probe manipulation and examination, considering other imaging modalities (e.g., epicardial echocardiography), obtaining gastroenterology consultation using a smaller probe, and using the most experienced operator [5, 12]. Unfortunately, we did not notice such a twisted distortion before the operation. Attempts to obtain the LV short-axis view would have been minimized or would not have been performed had we identified such a marked esophageal distortion before cardiac surgery. Preoperative evaluation of the course of the esophagus might provide information that is helpful for avoiding this life-threatening complication.

## Conclusion

We report a case of esophageal perforation after intraoperative TEE in a patient with a giant LA. Retrospectively constructed 3D images revealed an unusually distorted esophagus due to a giant LA that was possibly vulnerable to injury. A giant LA should be recognized as a risk factor for TEE-induced esophageal perforation.

## Abbreviations

3D: Three-dimensional
CPB: Cardiopulmonary bypass
CT: Computed tomography
EGJ: Esophagogastric junction
IABP: Intra-aortic balloon pumping
ICU: Intensive care unit
LA: Left atrium
LV: Left ventricular
MS: Mitral stenosis
NG: Nasogastric
POD: Postoperative day
TEE: Transesophageal echocardiography
